# Molecular System Bioenergetics—New Aspects of Metabolic Research

**DOI:** 10.3390/ijms10083655

**Published:** 2009-08-19

**Authors:** Valdur Saks

**Affiliations:** 1 INSERM U884, Laboratoire de Bioénergétique Fondamentale et Appliquée, Université Joseph Fourier 2280 Rue de la Piscine, BP 53, Grenoble Cedex 9, France; 2 Laboratory of Bioenergetics, National Institute of Chemical Physics and Biophysics, Akadeemia tee 23, 12618 Tallinn, Estonia

**Keywords:** molecular system bioenergetics, metabolism, systems biology, respiration, mitochondria

This Special Issue is a significant step in developing a new direction of metabolic research— Molecular System Bioenergetics, which itself is a part of Systems Biology. As a new paradigm of biological sciences, Systems Biology aims at understanding of biological functions by studies and description of new, system level properties, resulting from interactions between components of biological systems at any level of organization, from molecular to population. Metabolism is the way of life of cells by exchanging mass and energy with the surrounding medium, and understanding its mechanisms requires knowledge of the complex interactions between cellular systems and components. While studies of metabolism have a long history, new concepts of Systems Biology provide useful tools for metabolic research. According to Schrödinger, living cells need to be open systems with energy and mass exchange with the surrounding medium, with the aim of maintaining their high structural and functional organization and thus their internal entropy low, achieving this by means of increasing the entropy of the medium by catabolic reactions [1]. Thus, Schrödinger wrote: “The essential thing in metabolism is that the organism succeeds in freeing itself from all entropy it cannot help producing while alive” [1]. Thus, free energy conversion in the cells is an important, central part of metabolism, and understanding the complex mechanisms of its regulation is the aim of Molecular System Bioenergetics [2]. In this Special Issue, several important problems in this field were analyzed.

Two articles have focused on the historical roots and philosophical basis of Systems Biology [3,4]. These roots can be traced back as far as to the works of Harvey on circulation [3], to the discovery of homeostasis by Bernard [3,4], followed by creation of cybernetic theory of feedback regulation and information networks by Wiener, and the important and famous analysis of thermodynamic aspects of metabolism by Schrödinger [1] already mentioned above. In general, these developments and exponential growth of Systems Biology in recent years perfectly fit with the Hegel’s philosophical predictions of dialectic developments of historical processes when applied to biology [4].

The next two articles deal with experimental and theoretical methods of studies of interactions between cellular components [5,6]. One of them [5] describes two-hybrid methods of studies of protein-protein interactions [5], and the second deals with theoretical basis and experimental analysis of metabolic fluxes by isotopomer analysis [6]. Then, the following chapter deals with experimental analysis of phosphotransfer networks [7]. These analyses may provide a good basis for further mathematical modelling of organized metabolic systems.

After these chapters of general interest, the focus of interest of authors is fixed on mitochondria— on the energetic factories of the cells. One of the most important directions of the bioenergetics of the cells *in vivo* are studies of mitochondrial organization and dynamics by confocal microscopy and image analysis, as described in [8], and in [9] new evidence is given for the existence of a novel mitochondrial potassium channel. This is followed by a mathematical analysis of mitochondrial functional behavior *in vivo* by applying the methods of Metabolic Control Analysis in [10].

The last group of articles, [11–14], deals with an important application of Molecular System Bioenergetics—mitochondrial pathologies. These papers describe the phenomenon of energetic depression [11], the application of the system biology approach in studies of apoptosis [12], in cancer research [13] and finally, in obesity and ageing [14].

Taken together, all articles published describe well the most interesting current directions in cellular energetics and metabolism studies. It is a pleasure of the Guest Editor to thank all authors for their important contributions into this Special Issue and development of the new direction of Molecular System Bioenergetics.
